# Supplementary material for: TgAP2IX-5 is a key transcriptional regulator of the asexual cell cycle division in Toxoplasma gondii
Source: Nat Commun. 2021 Jan 7;12:116. doi: 10.1038/s41467-020-20216-x (PMC7791101; doi:10.1038/s41467-020-20216-x)
Supplement: Supplementary file 1 — Supplementary Information [file 41467_2020_20216_MOESM1_ESM.docx]

**Supplementary information**

**TgAP2IX-5 is a key transcriptional regulator of the asexual cell cycle division in *Toxoplasma gondii***

Asma S. Khelifa ^1^, Cecilia Guillen Sanchez ^1^, Kevin M. Lesage ^1^, Ludovic Huot ^1^, Thomas Mouveaux ^1^, Pierre Pericard ^2^, Nicolas Barois ^1^, Helene Touzet ^2,3^, Guillemette Marot ^2,4^, Emmanuel Roger ^1^ and Mathieu Gissot ^1,*^.

1. Univ. Lille, CNRS, Inserm, CHU Lille, Institut Pasteur de Lille, U1019 - UMR 9017 - CIIL - Center for Infection and Immunity of Lille, F-59000 Lille, France.
2. Univ. Lille, CNRS, Inserm, CHU Lille, Institut Pasteur de Lille, US 41 - UMS 2014 - PLBS, bilille, F-59000 Lille, France.
3. Univ. Lille, CNRS, Centrale Lille, UMR 9189 - CRIStAL - Centre de Recherche en Informatique Signal et Automatique de Lille, F-59000 Lille, France.
4. Univ. Lille, Inria, CHU Lille, ULR 2694 - METRICS: Evaluation des technologies de santé et des pratiques médicales, F-59000 Lille, France.

* Corresponding author: [mathieu.gissot@pasteur-lille.fr](mailto:mathieu.gissot@pasteur-lille.fr)

**This file includes:**

Supplementary Figures 1 to 11

Supplementary Table 1

**
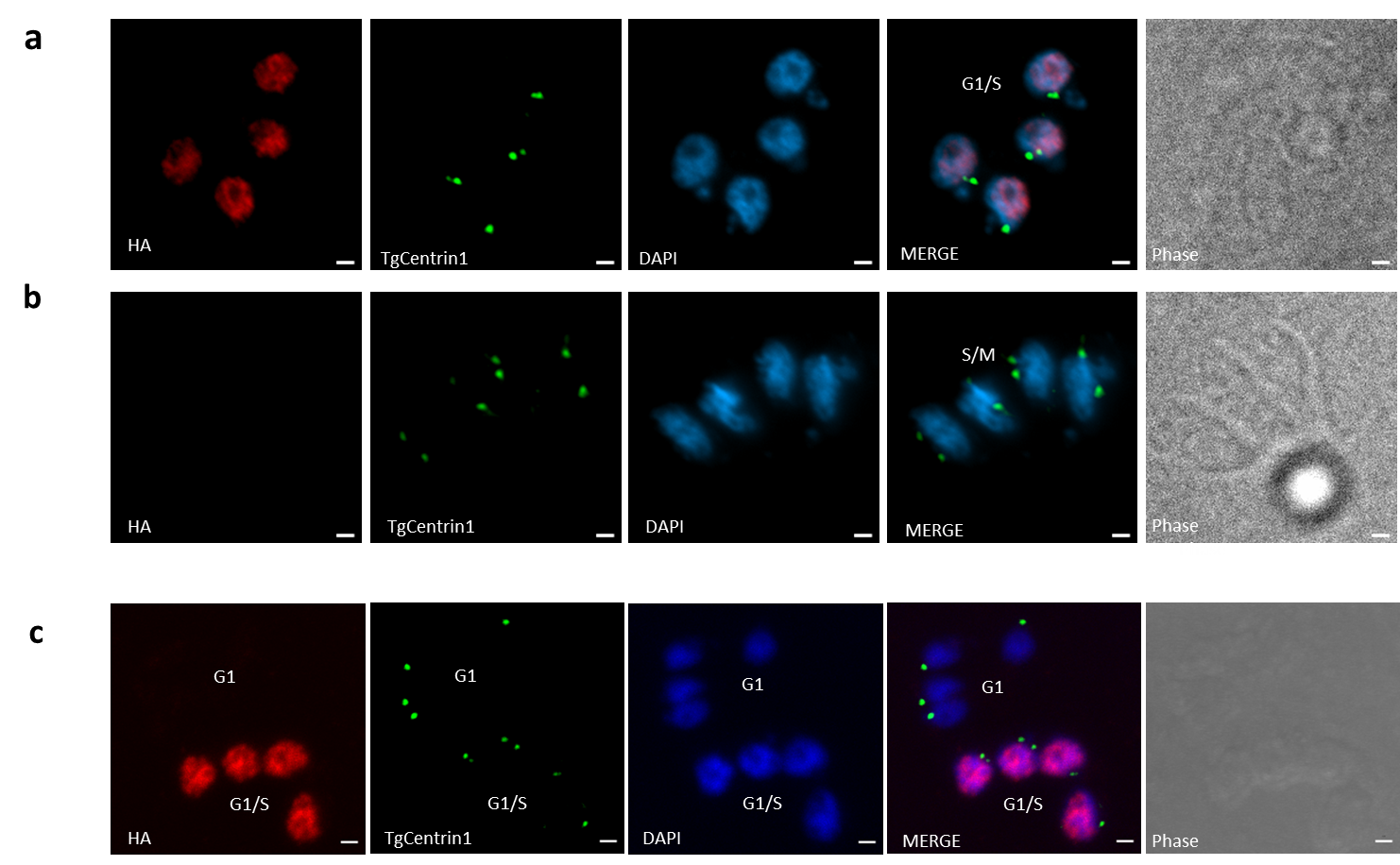
**

**Supplementary Figure 1**. **Cell cycle expression of the TgAP2IX-5 protein.**

Confocal imaging demonstrating the expression of TgAP2IX-5 protein during different stages of the tachyzoite asexual cell cycle using anti-TgCentrin1 as a marker of the cell cycle. Expression of TgAP2IX-5 is indicated in red (HA-tag) and TgCentrin1 is indicated in green.DAPI was used to stain the nucleus. Scale bar is indicated at the lower right side of each image. (**a**) TgAP2IX-5 expression during the G1/S phase. (**b**) TgAP2IX-5 expression during the S/M phase. (**c**) TgAP2IX-5 expression of parasites within two separate vacuoles at different stages of the cell cycle G1/S and G1.

**
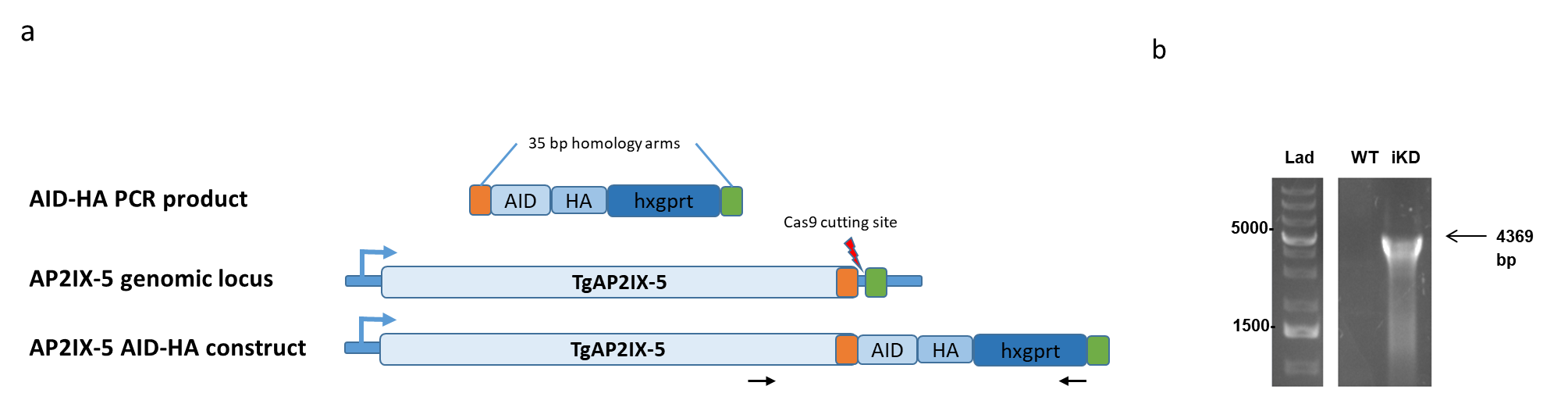
**

**Supplementary Figure 2. Production of a iKD TgAP2IX-5 mutant strain.**

(**a**) Diagram showing the strategy used to generate *Toxoplasma gondii* parasites expressing AP2IX-5 tagged with AID-HA-HXGPRT at the C terminus. The location of the primers used for integration PCR is indicated by arrows. (**b**) PCR confirming the insertion of AID-HA-HXGPRT insert at the endogenous locus coding for TgAP2IX-5.

**
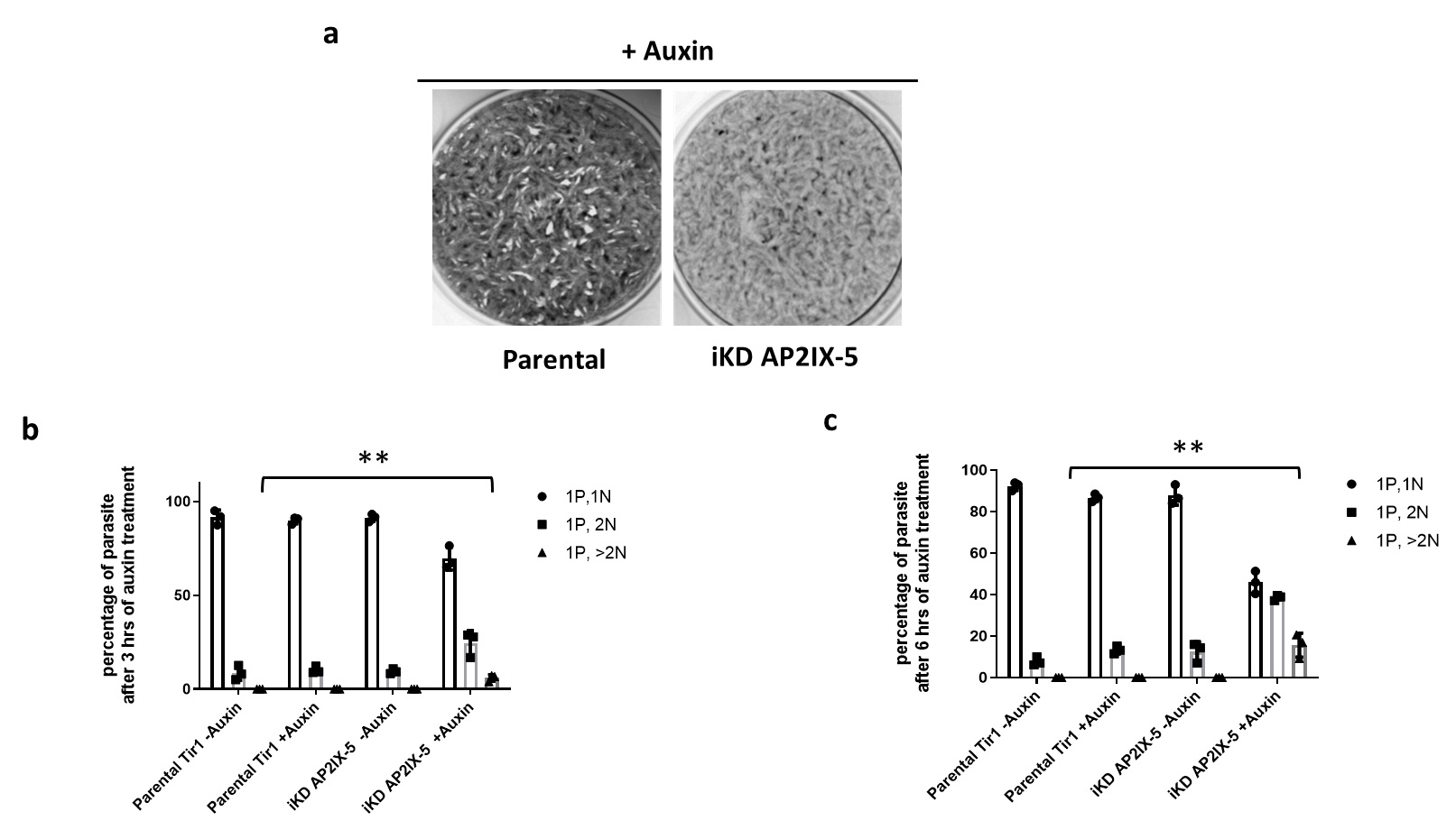
**

**Supplementary Figure 3. Phenotype of the iKD** **TgAP2IX-5 strain.**

(**a**) Plaque assay for the parental and iKD TgAP2IX-5 strains in the presence of auxin treatment for 7 days. (**b**) Bar graph representing nucleus per parasite counts for parental Tir1 and iKD TgAP2IX-5 strains in the absence and presence of 3 hours of auxin treatment. AStudent’s t-test was performed to compare between the mean percentage of multinucleated parasites between the control (Parental Tir1 -auxin) and the iKD TgAP2IX-5 mutant strain. Two-sided p-values: **: p=0.0049; mean ± s.d. (n=3 independent experiments). (**c**) Bar graph representing nucleus per parasite counts for the parental and iKD TgAP2IX-5 strains in the absence and presence of 6 hours of auxin treatment. Student’s t-test was performed to compare between the mean percentage of multinucleated parasites between the control (Parental Tir1 -auxin) and the iKD TgAP2IX-5 mutant strain. Two-sided p-values: ** P=0.0097; mean ± s.d. (n=3 independent experiments).

**
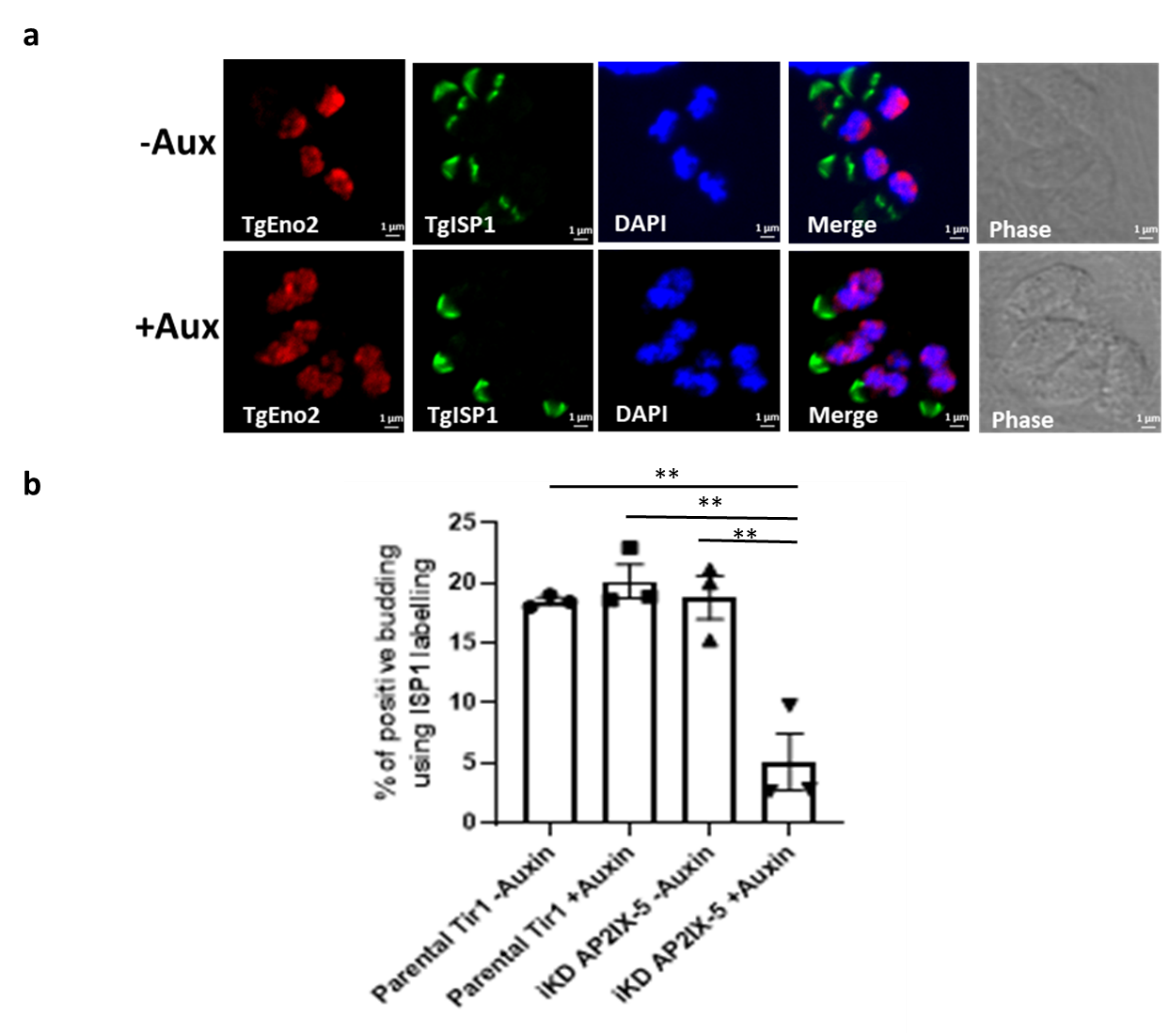
**

**Supplementary Figure 4**. **Production of daughter cells is severely impaired in absence of TgAP2IX-5**

(**a**) Confocal imaging of iKD TgAP2IX-5 labelled with TgEno2 (red) and TgISP1 (green) in the presence and absence of auxin treatment. DAPI was used to stain the nucleus. Scale bar is indicated at the lower right side of each image. (**b**) Bar graph representing the percentage of daughter parasite formation in the absence and presence of 6 hours of auxin treatment 24 hours post-infection using TgISP1labelling, A Student’s t-test was performed, Two-sided p-values: **: p=0.0048 (iKD TgAP2IX-5 +auxin compared to Parental Tir1 – auxin), **: p=0.0054 (iKD TgAP2IX-5 +auxin compared to Parental Tir1 +auxin), **: p=0.0098 (iKD TgAP2IX-5 +auxin compared to iKD TgAP2IX-5 -auxin); mean ± s.d. (n=3 independent experiments).

**
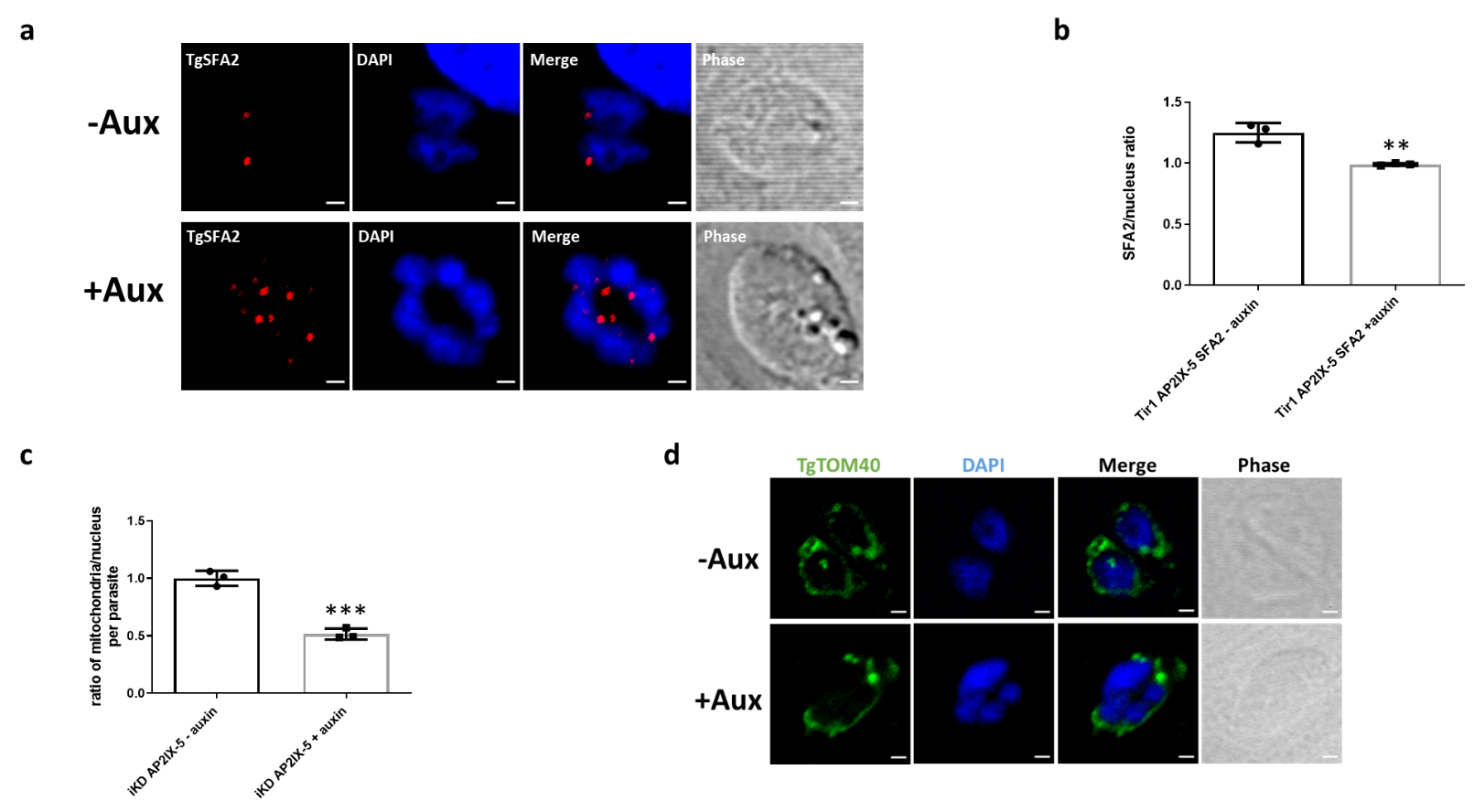
**

**Supplementary Figure 5. Effect of TgAP2IX-5 on TgSFA2 and mitochondrion replication in absence of TgAP2IX-5.**

**(a)** Confocal imaging of iKD AP2IX-5 TgSFA2-myc. TgSFA2 is labelled in red and the nucleus is stained with DAPI. Scale bar is indicated in the lower right of each image. **(b)** Bar graph representing the ratio of SFA2: nucleus in the absence and presence of overnight auxin treatment. A Student’s t-test was performed, Two-sided p-values: **: p=0.0017; mean ± s.d. (n=3 independent experiments). **(c)** Bar graph representing the ratio of mitochondria: nucleus in the absence and presence of overnight auxin treatment. A Student’s t-test was performed; two-sided p-values: ***: p=0.0005; mean ± s.d. (n=3 independent experiments). **(d)** Confocal imaging of iKD TgAP2IX-5 labelled with TgTom40 (mitochondria) in green in the presence and absence of overnight auxin treatment. DAPI was used to stain the nucleus. Scale bar is indicated in the lower right side of each image.

**
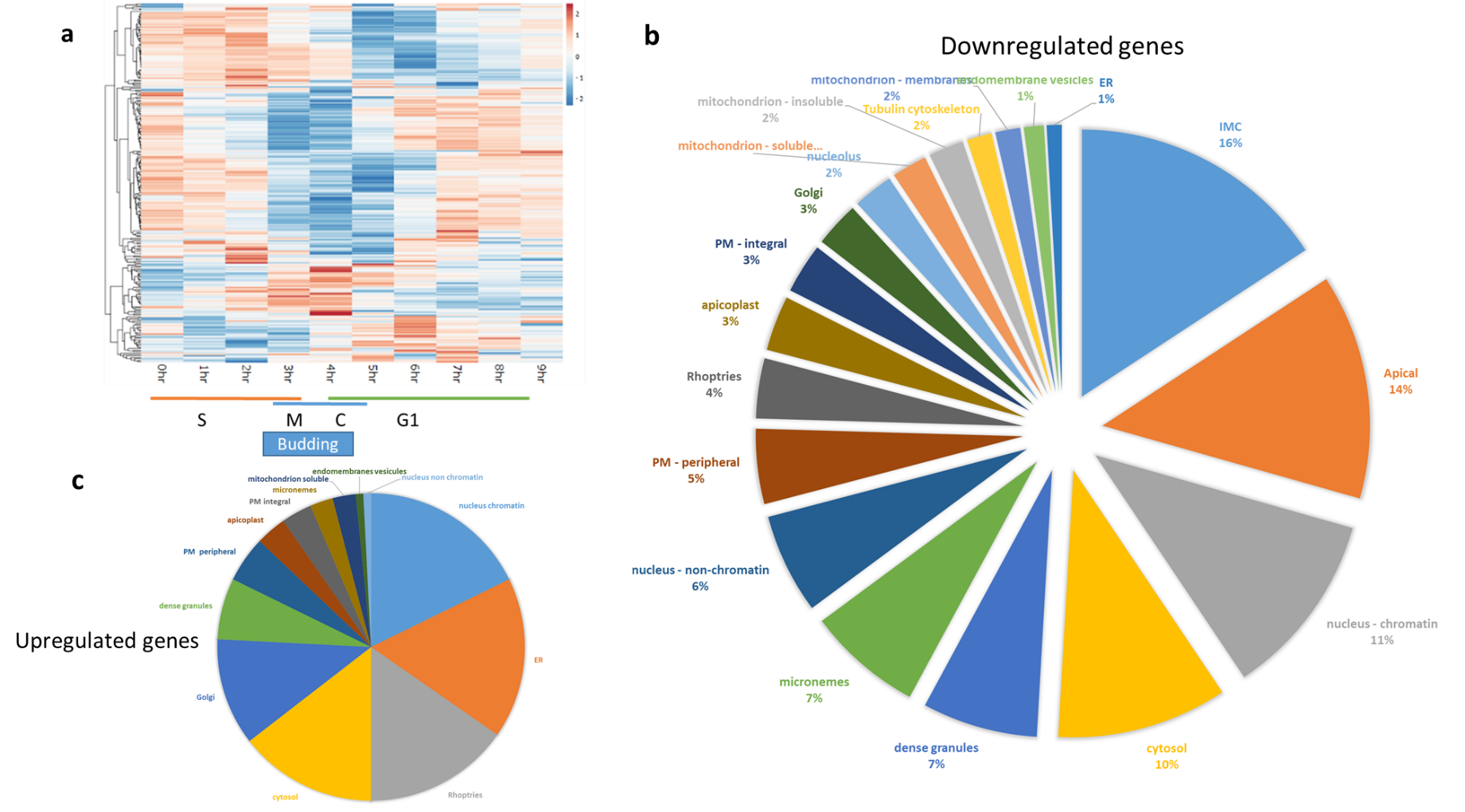
**

**Supplementary Figure 6. Cell cycle expression and putative localization of differentially expressed genes.**

**(a)** Heat map of the cell cycle expression profile for all individual transcripts that are upregulated in the iKD AP2IX-5 strain in the presence of 6 hours of auxin treatment. Scale of expression is color-coded with highly expressed genes in orange and less expressed genes in blue according to cell cycle phase indicated at the bottom of the heat map (S-M-C-G1). The cell cycle phases are represented at the bottom as well as the timing when budding occurs. Although the majority of up-regulated genes are not expressed in S/M phase, a cluster of genes shows a strong expression during this phase. **(b)** Pie chart representing the distribution of the putative localization, according to Barylyuk *et al.*, of the proteins encoded by downregulated transcripts. **(c)** Pie chart representing the distribution of the putative localization, according to Barylyuk *et al.*, of the proteins encoded by upregulated transcripts.

**
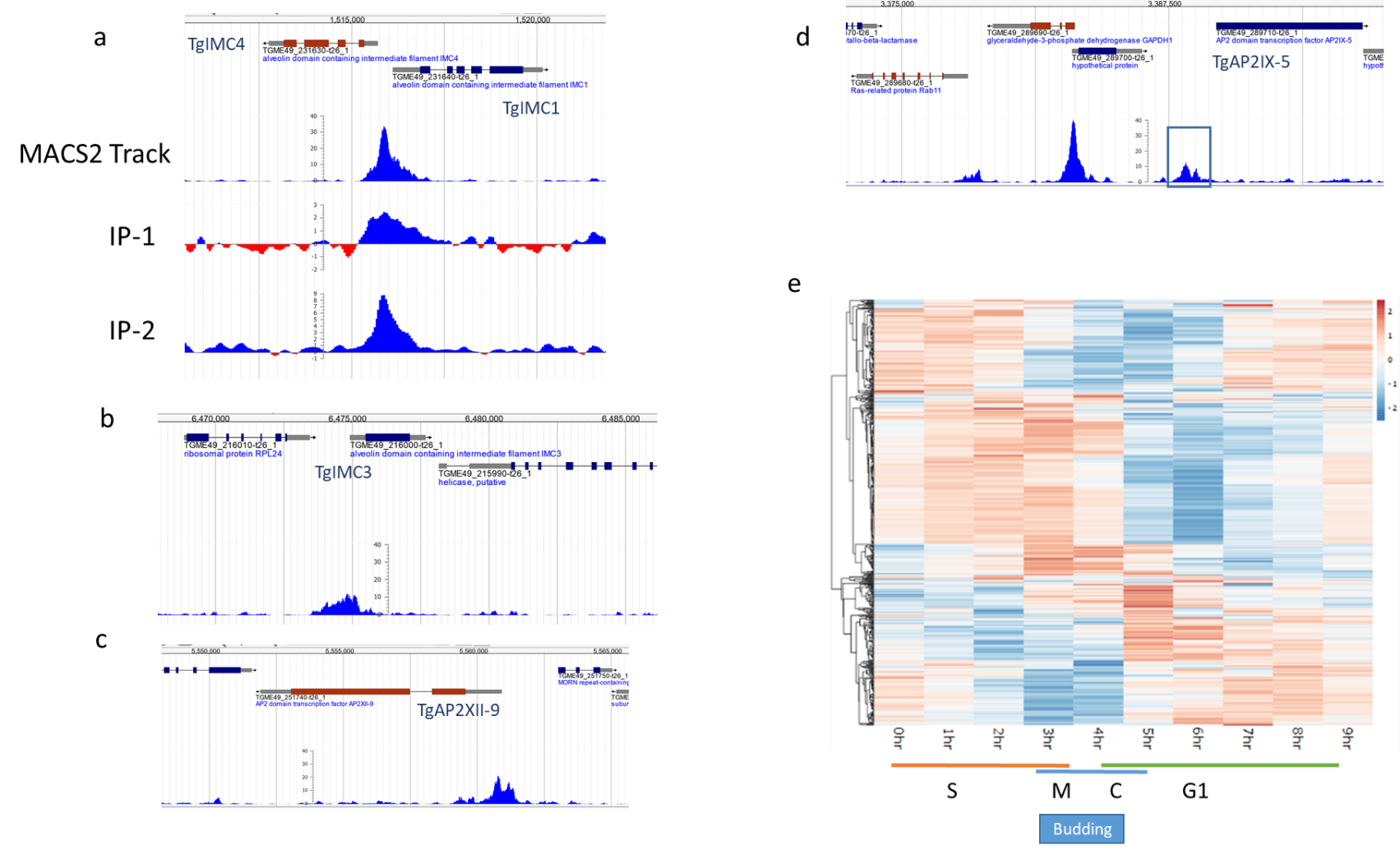
**

**Supplementary Figure 7.** **ChIP-seq data analysis represented by peaks targeting the promoter of several genes**.

(**a**) MACS2 generated track and individual ChIP tracks (background subtracted) representing the direct targeting of TgAP2IX-5 to the promoter of downregulated gene TgIMC1 and TgIMC4. (**b**) MACS2 track representing the direct targeting of TgAP2IX-5 to the promoter of downregulated gene TgIMC3. **(c)** MACS2 track representing the direct targeting of TgAP2IX-5 to the promoter of downregulated gene TgAP2XII-9. (**d**) ChIP-seq data peak indicated by blue box demonstrating the targeting of TgAP2IX-5 towards upregulated gene TgAP2IX-5 (boxed). (**e**) Heat map representing the cell cycle expression of all individual transcripts that are targeted by TgAP2IX-5 based on ChIP-seq analysis. Phases of the cell cycle are indicated at the bottom of the figure. The approximate timing for the budding cycle is indicated at the bottom.

**
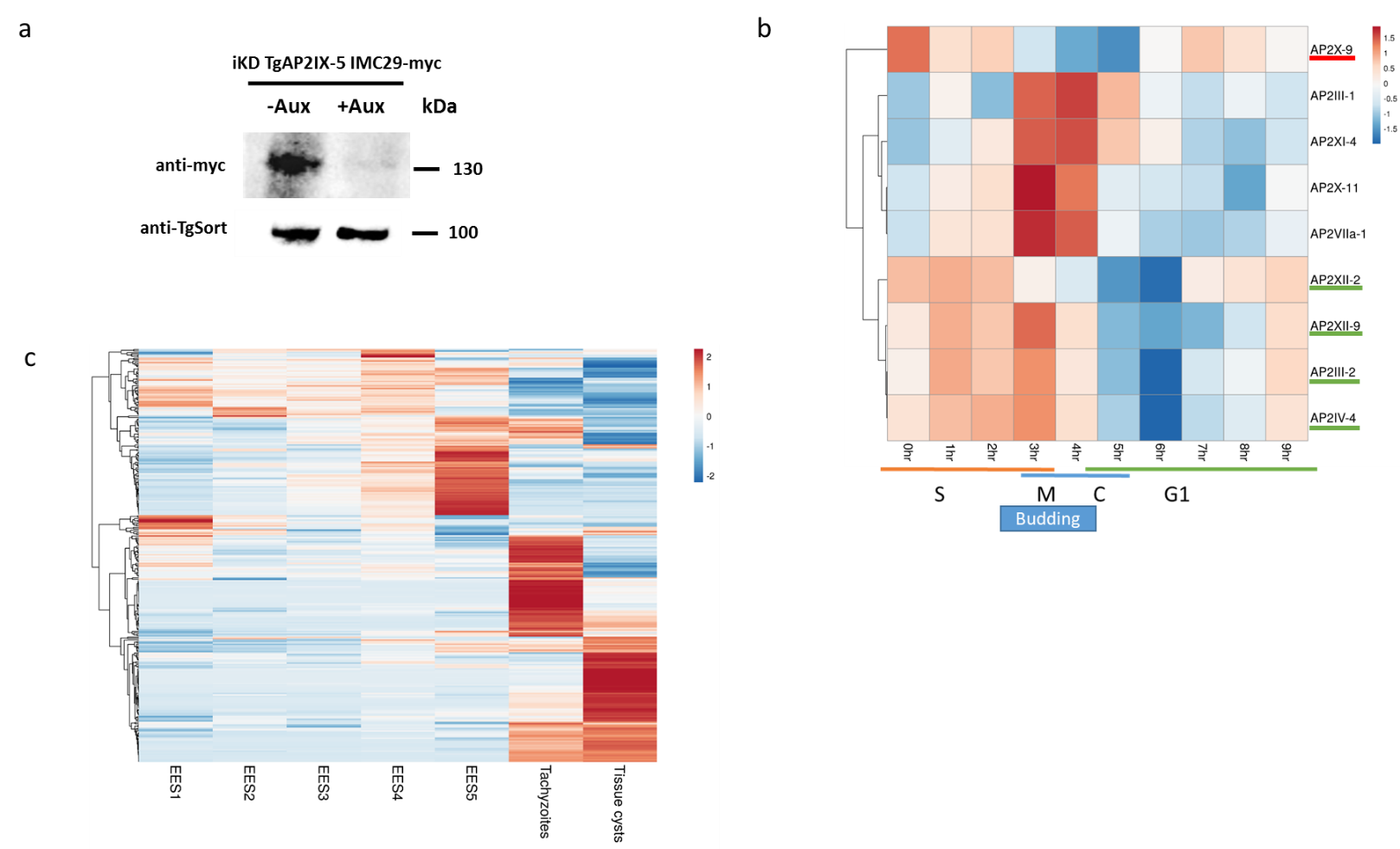
**

**Supplementary Figure 8. Characterisation of genes differentially regulated in the iKD TgAP2XI-5 mutant.**

(**a**) Western blot depicting the total protein extract of iKD TgAP2IX-5 IMC29-myc strain treated with or without auxin for 24 hours. Western blots were probed with anti-myc to detect the presence of the TgIMC29 protein (upper panel), anti-TgSortilin was used as a control for normalization (lower panel).​ (**b**) Heat map of 9 individual TgApiAP2 TF transcripts that are downregulated and upregulated during 6 hours of TgAP2IX-5 depletion. Cell cycle phases are indicated at the lower bottom (S-M-C-G1). Downregulated ApiAP2 TF that are directly bound to promoters are underlined. The downregulated ApiAP2 TFs are underlined in green. The upregulated ApiAP2 TF is underlined in red. (**c**) Heat map of upregulated transcripts that are expressed during the parasite life cycle (cat sexual stages, tachyzoite and bradyzoite stages). EES stands for enteroepithelial developmental stages.


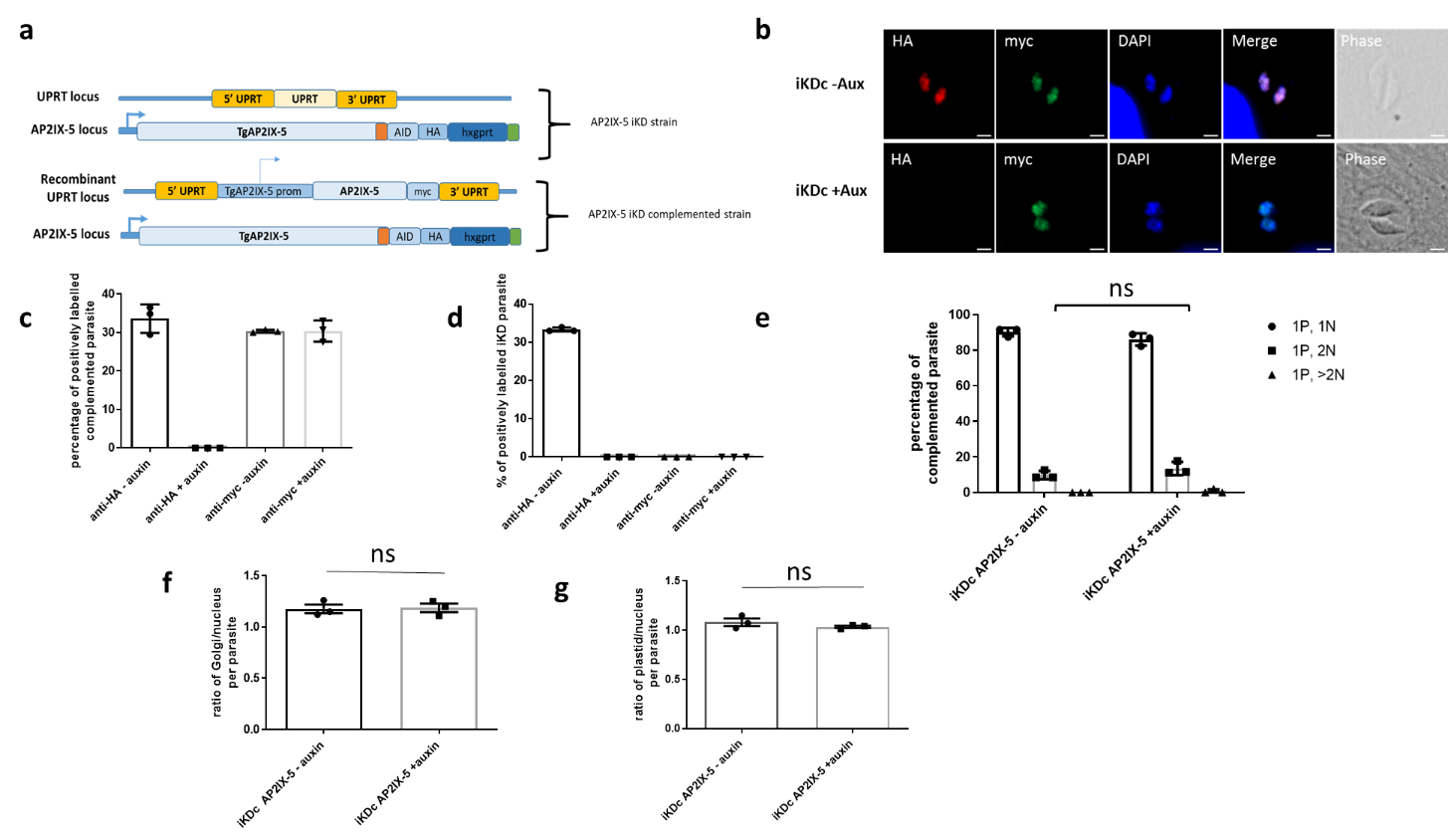


**Supplementary Figure 9.** **Complementation of the iKD TgAP2IX-5 demonstrate that the TgAP2IX-5 protein is responsible for the phenotypes observed in the mutant.**

(**a**) TgAP2IX-5 iKD complementation schematic representation demonstrating the strategy used for generating the complemented TgAP2IX-5 iKD strain by targeting the UPRT locus and replacing it with exogenous myc-tagged TgAP2IX-5 under the control of its own specific promoter. (**b**) Confocal imaging of ciKD TgAP2IX-5 strain. Endogenous TgAP2IX-5 tagged with HA is represented in red while exogenous TgAP2IX-5 tagged with myc is represented in green. (**c**) Bar graph representing the expression of TgAP2IX-5 using anti-HA and anti-myc antibodies in the complemented strain. mean ± s.d. (n=3 independent experiments). (**d**) Bar graph representing the expression of iKD TgAP2IX-5 using anti-HA antibody and anti-myc antibody as a negative control; mean ± s.d. (n=3 independent experiments). (**e**) Bar graph representing nucleus per parasite counts in the iKDc TgAP2IX-5 strain in the presence and absence of overnight auxin treatment. A Student’s t-test was performed to compare mean percentage of multinucleated parasite in the iKDc TgAP2IX-5 strain (-auxin) and iKDc TgAP2IX-5 strain (+auxin). Two-sided p-values: ns: p=0.3739; mean ± s.d. (n=3 independent experiments). (**f**) Bar graph representing Golgi: nucleus ratio in the iKDc TgAP2IX-5 in the presence and absence of overnight auxin treatment. A Student’s t-test was performed, two-sided p-values: ns: p=0.9387; mean ± s.d. (n=3 independent experiments). (**g**) Bar graph representing plastid: nucleus ratio in the iKDc TgAP2IX-5 in the presence and absence of overnight auxin treatment. A Student’s t-test was performed, two-sided p-values: ns: p=0.1630; mean ± s.d. (n=3 independent experiments).

**
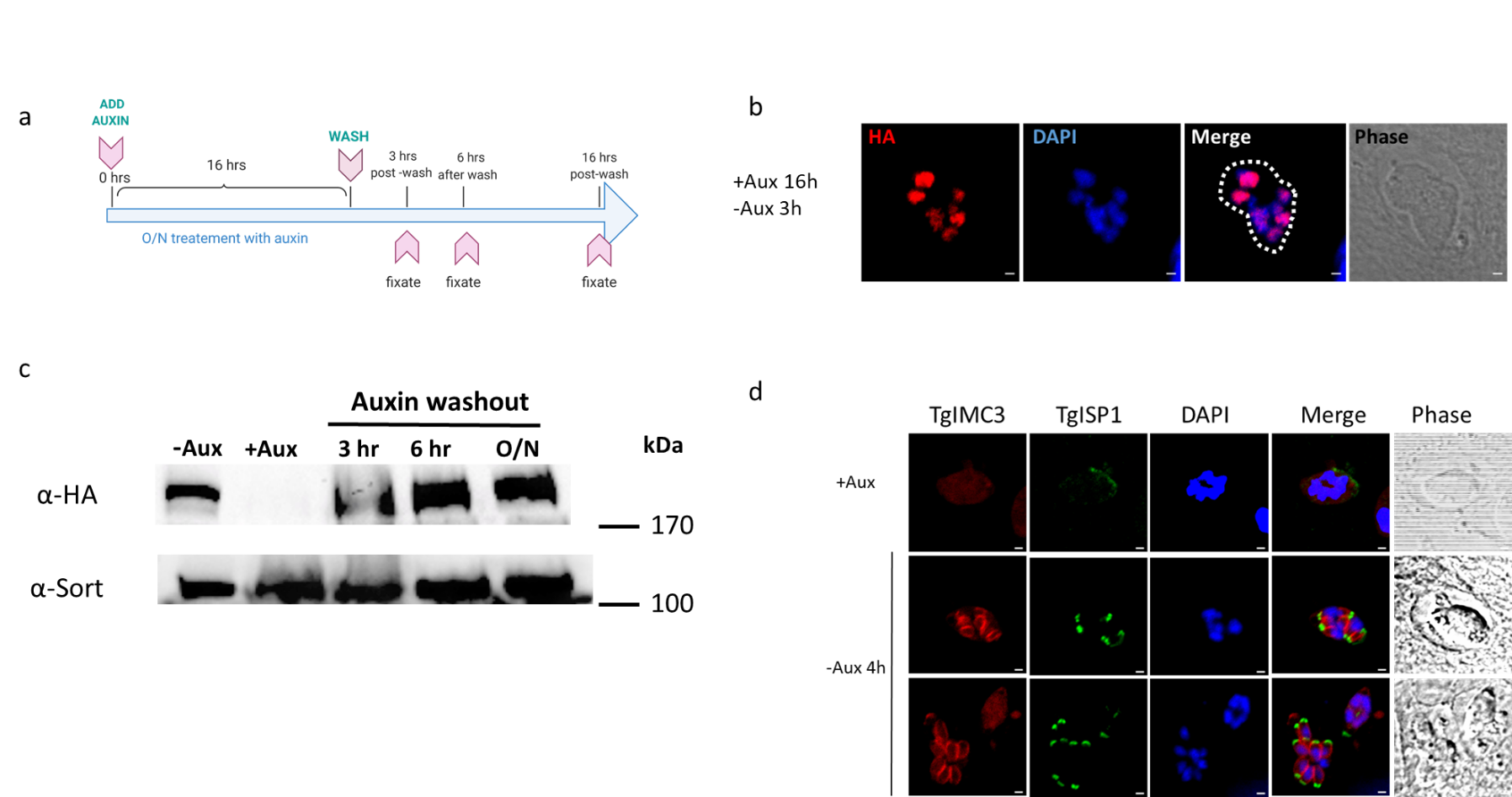
**

**Supplementary Figure 10. Auxin washout allows the re-expression of TgAP2IX-5**

**(a)** Schematic representation of auxin treatment and washout times used to carry out re-expression experiments of TgAP2IX-5. (**b**) Immunofluorescence assay of iKD TgAP2IX-5 parasites treated with auxin for 16 hours and re-expressing TgAP2IX-5 after auxin washout and culture without auxin for 3 hours. TgAP2IX-5 is indicated in red (HA-tagged). DAPI was used to stain the nucleus. Scale bar is indicated in the lower right side of each image. (**c**) Western-blot depicting the total protein extract of iKD TgAP2IX-5-HA strain treated with (+Aux) or without (-Aux) auxin for 16 hours. Parasites extracts were also produced after a 16 hours auxin treatment and then cultured for 3h, 6h or over-night without auxin (Aux washout, 3h, 6h and O/N, respectively). Western blots were probed with anti-HA to detect the presence of the TgAP2IX-5 protein (upper panel), anti-TgSortilin was used as a control for normalization (lower panel). (**d**) Immunofluorescence assays of iKD TgAP2IX-5 parasites before auxin washout and after auxin washout for a duration of 4 hours. TgIMC3 is labelled in red. TgISP1 is labelled in green. DAPI was used to stain the nucleus. Scale bar is indicated at the lower right side of each image.


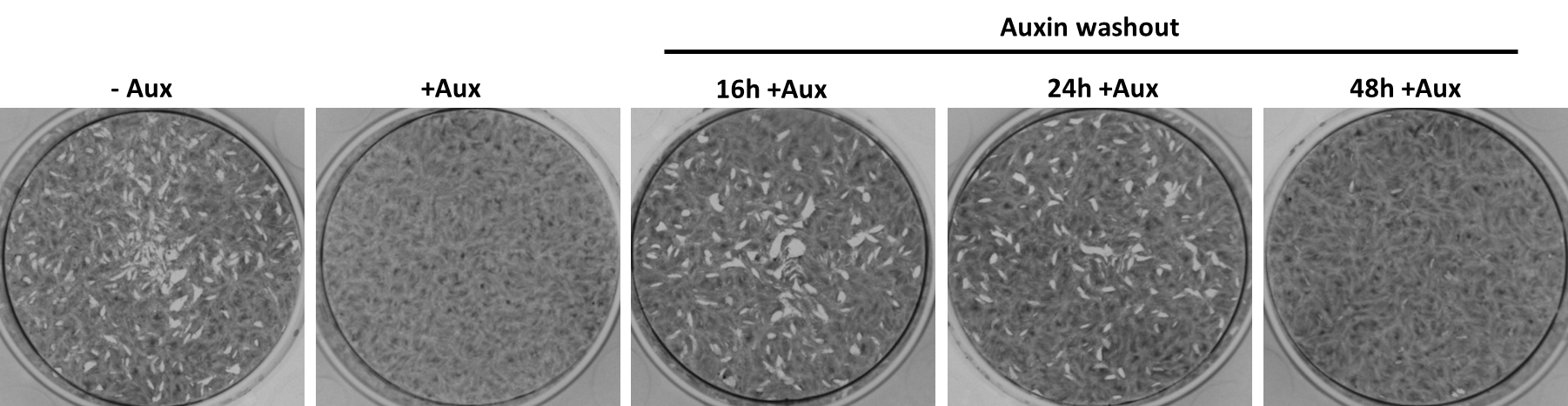


**Supplementary Figure 11**. **Parasites are viable after a cycle of forced endopolygeny**.

Plaque assay images representing the viability of iKD TgAP2IX-5 parasites after auxin washout. Parasites were treated with auxin for 16 hours, 24 hours, and 48 hours prior to washout.

**Supplementary Table 1:** Oligonucleotides used in this study.

| **Primer name** | **TOXODB Gene ID** | **Sequence** |
| --- | --- | --- |
| TgAP2IX-5iKD forward | TGME49_289710 | 5'-GGTACGGACGAAACCAAGTTGACGACTCCAGTGGATATCGGgctagcAAGGGCTCGG-3' |
| TgAP2IX-5 iKD reverse | TGME49_289710 | 5'-CTTCTGTGTCCATTTCTCCCCTTGGCTCCCGAGACCCCTTTGAATACGACTCACTATAGG-3' |
| 3' Cas9 TgAP2IX-5 | TGME49_289710 | 5'-GGAGCGTAGAAAAGAAAGCGGTTTTAGAGCTAGAAATAGCAAGTTAAA-3' |
| TgSFA2 KI forward | TGME49_205670 | 5'-CCTTACAGAAGGGCCTTCGAAATATAACAAGTCGCAAAATTGGAAGTGGAGGACGG-3' |
| TgSFA2 KI reverse | TGME49_205670 | 5'-ATACCGTGCTGATTCTTCTCTGAGAATCATCAACACGAATTGGAGCTCCACCGC-3' |
| 3' Cas9 TgSFA2 | TGME49_205670 | 5'-CGTACTGCACGCCCATGCAAGTTTTAGAGCTAGAAATAGCAAGTTAAA-3' |
| TgIMC29 KI forward | TGME49_243200 | 5'-CGCTCAGGCAGCAGTACCCCGGACACGGCCTCAATAAAATTGGAAGTGGACGG-3' |
| TgIMC29 KI reverse | TGME49_243200 | 5'-GTCACATCCGTATACACGGTTCTATGAGCAGATTCCGAATTGGAGCTCCACCGC-3' |
| 3' Cas9 TgIMC29 | TGME49_243200 | 5'-AGCAGCATGATTACGTATTCGTTTTAGAGCTAGAAATAGCAAGTTAAA-3' |
