# Supplementary material for: TgAP2IX-5 is a key transcriptional regulator of the asexual cell cycle division in Toxoplasma gondii
Source: Nat Commun. 2021 Jan 7;12:116. doi: 10.1038/s41467-020-20216-x (PMC7791101; doi:10.1038/s41467-020-20216-x)
Supplement: Supplementary file 3 — Description of Additional Supplementary Files [file 41467_2020_20216_MOESM3_ESM.pdf]

Description of Additional Supplementary Files

File Name: Supplementary Data 1

Description: RNASeq results. Summary of all genes up or downregulated with a fold change above (-1 or 1 of Log2 fold change) and a FDR of 0.05. The following information is provided: gene accession number; gene annotation (Product description); Log2 fold change (DESeq2 log2 FC); putative localization in the parasite (Localization LOPIT); presence of the gene in the ChIP dataset (ChIP; gene accession number= presence, #N/A=absence).

File Name: Supplementary Data 2

Description: ChIP-seq results. Gene IDs of the promoters bound by TgAP2IX-5 as identified by MACS2 with a FDR of 0.05. The following information is provided: gene accession number; gene annotation (Product description); putative localization in the parasite (Localization LOPIT); presence of the gene in the RNA-seq dataset (Presence in the RNA-seq dataset; gene accession number = presence, #N/A=absence).

File Name: Supplementary Movie 1.

Description:: Video-microscopy of the iKD TgAP2IX-5 parasite after auxin washout. The IMC of the parasite is labelled with IMC3-mCherry. Each image is taken at 13 minutes intervals.
