# Supplementary material for: TgAP2IX-5 is a key transcriptional regulator of the asexual cell cycle division in Toxoplasma gondii
Source: Nat Commun. 2021 Jan 7;12:116. doi: 10.1038/s41467-020-20216-x (PMC7791101; doi:10.1038/s41467-020-20216-x)
Supplement: Supplementary file 7 — Reporting Summary [file 41467_2020_20216_MOESM7_ESM.pdf]

nature research

Corresponding author(s): Gissot Mathieu

Last updated by author(s): Nov 2, 2020

Reporting Summary

Nature Research wishes to improve the reproducibility of the work that we publish. This form provides structure for consistency and transparency in reporting. For further information on Nature Research policies, see our Editorial Policies and the Editorial Policy Checklist.

Statistics

For all statistical analyses, confirm that the following items are present in the figure legend, table legend, main text, or Methods section.

n/a	Confirmed	
☐	☒	The exact sample size (*n*) for each experimental group/condition, given as a discrete number and unit of measurement
☐	☒	A statement on whether measurements were taken from distinct samples or whether the same sample was measured repeatedly
☐	☒	The statistical test(s) used AND whether they are one- or two-sided<br>*Only common tests should be described solely by name; describe more complex techniques in the Methods section.*
☒	☐	A description of all covariates tested
☒	☐	A description of any assumptions or corrections, such as tests of normality and adjustment for multiple comparisons
☐	☒	A full description of the statistical parameters including central tendency (e.g. means) or other basic estimates (e.g. regression coefficient) AND variation (e.g. standard deviation) or associated estimates of uncertainty (e.g. confidence intervals)
☐	☒	For null hypothesis testing, the test statistic (e.g. *F*, *t*, *r*) with confidence intervals, effect sizes, degrees of freedom and *P* value noted<br>*Give P values as exact values whenever suitable.*
☒	☐	For Bayesian analysis, information on the choice of priors and Markov chain Monte Carlo settings
☒	☐	For hierarchical and complex designs, identification of the appropriate level for tests and full reporting of outcomes
☒	☐	Estimates of effect sizes (e.g. Cohen's *d*, Pearson's *r*), indicating how they were calculated

Our web collection on statistics for biologists contains articles on many of the points above.

Software and code

Policy information about availability of computer code

Data collection	Clusters were generated on a flow cell with within a cBot2 using the Cluster Generation Kit (Illumina). Libraries were sequenced as 50 bp-reads on a HiSeq 2500 using the sequence by synthesis technique (Illumina).
Data analysis	RNA-seq and ChIP-seq raw reads were quality-checked, pre-processed and cleaned with FastQC v0.11.8-0, Cutadapt v1.18 and Trimmomatic v0.38.1<br>RNA-seq cleaned reads were aligned with HiSAT2 v2.1.0, genes expression was quantified with htseq-count from the HTSeq suite v0.9.1. Differential expression analysis was performed using DESeq2 v1.22.1 within the SARTools framework v1.6.6.<br>ChIP-seq cleaned reads were aligned with Bowtie2 v2.3.4. Alignments were merged with SAMtools v1.9, duplicates identified with Picard MarkDuplicates v2.18.20. ChIP quality was checked with deepTools suite v3.1.3. Peak calling performed with MACS2 v2.1.2.<br>GraphPad Prism 8 was used for statistical analysis and graphical representation.<br>Microscopy: Carl Zeiss Zen2

For manuscripts utilizing custom algorithms or software that are central to the research but not yet described in published literature, software must be made available to editors and reviewers. We strongly encourage code deposition in a community repository (e.g. GitHub). See the Nature Research guidelines for submitting code & software for further information.

Data

Policy information about availability of data

All manuscripts must include a data availability statement. This statement should provide the following information, where applicable:

- Accession codes, unique identifiers, or web links for publicly available datasets
- A list of figures that have associated raw data
- A description of any restrictions on data availability

RNA-seq and ChIP-seq data that support the findings of this study have been deposited in GEO database under the accession number GSE150406 (https://www.ncbi.nlm.nih.gov/geo/query/acc.cgi?acc=GSE150406). Source data are provided with this paper.

Field-specific reporting

Please select the one below that is the best fit for your research. If you are not sure, read the appropriate sections before making your selection.

☒ Life sciences ☐ Behavioural & social sciences ☐ Ecological, evolutionary & environmental sciences

For a reference copy of the document with all sections, see nature.com/documents/nr-reporting-summary-flat.pdf

Life sciences study design

All studies must disclose on these points even when the disclosure is negative.

Sample size	Sample size were chosen based on previously published litterature for the performed assays (1). All experiments were performed with enough biological replicates (a minimum of 3) to allow relevant statistical analysis.<br>1. Suvorova, E. S., Francia, M., Striepen, B. & White, M. W. A novel bipartite centrosome coordinates the apicomplexan cell cycle. PLoS Biol. 13, e1002093 (2015).
Data exclusions	No data were excluded.
Replication	All experiments were done with biological replicates and take into account the biological variation of the system. At least three biological replicates and two technical replicates were performed. All results were succefully replicated.
Randomization	Assignement of strains to treatment group were randomized in the study.
Blinding	Investigators were not blinded during the experiments. The analysis perfomed have quantitative endpoints and are not subjected to investigator bias.

Reporting for specific materials, systems and methods

We require information from authors about some types of materials, experimental systems and methods used in many studies. Here, indicate whether each material, system or method listed is relevant to your study. If you are not sure if a list item applies to your research, read the appropriate section before selecting a response.

Materials & experimental systems

n/a	Involved in the study	
☐	☒	Antibodies
☒	☐	Eukaryotic cell lines
☒	☐	Palaeontology and archaeology
☒	☐	Animals and other organisms
☒	☐	Human research participants
☒	☐	Clinical data
☒	☐	Dual use research of concern

Methods

n/a	Involved in the study	
☐	☒	ChIP-seq
☒	☐	Flow cytometry
☒	☐	MRI-based neuroimaging

Antibodies

Antibodies used	Primary antibodies used for IFAs include: anti-TgEno2, anti-TgISP1, anti-TgIMC1 (a gift from Pr. Ward, U. Vermont), anti-TgCentrin1 (a gift from Pr. Gubbels, Boston College), anti-TgChromo1, anti-TgSortilin (Golgi), anti-TgACP (Plastid), anti-TgTom40 [3] (mitochondrion), anti-HA (Roche, #11867423001) and anti-myc (abcam, #ab10910) antibodies were used at the following dilutions: 1:1000, 1:500, 1:500, 1:500, 1:500, 1:500, 1:500, 1:500, 1:200, respectively.<br>Secondary antidodies for IFAs include: Anti-rabbit Alexa-fluor 488 (Thermo-Fisher, cat # A-11008). Anti-rabbit Alexa-fluor 594 (Thermo-Fisher, cat # A-11012). Anti-mouse Alexa-fluor 488 (Thermo-Fisher, cat # A-11001). Anti-mouse Alexa-fluor 594 (Thermo-Fisher, cat # A-11005). Anti-rat Alexa-fluor 488 (Thermo-Fisher, cat # A-11001). Anti-rat Alexa-fluor 594 (Thermo-Fisher, cat # A-11006).

Validation

The antibodies were validated in the following publications:
anti-TgEno2 : Dzierszinski F, Mortuaire M, Dendouga N, Popescu O, Tomavo S. Differential expression of two plant-like enolases with distinct enzymatic and antigenic properties during stage conversion of the protozoan parasite Toxoplasma gondii. J Mol Biol. 2001;309:1017–27.
anti-TgISP1: Beck JR, Rodriguez-Fernandez IA, Leon JC de, Huynh M-H, Carruthers VB, Morrissette NS, et al. A Novel Family of Toxoplasma IMC Proteins Displays a Hierarchical Organization and Functions in Coordinating Parasite Division. PLOS Pathog. Public Library of Science; 2010;6:e1001094.
anti-TgIMC1 (a gift from Pr. Ward, U. Vermont): Wichroski MJ, Melton JA, Donahue CG, Tweten RK, Ward GE. Clostridium septicum alpha-toxin is active against the parasitic protozoan Toxoplasma gondii and targets members of the SAG family of glycosylphosphatidylinositol-anchored surface proteins. Infect Immun. 2002;70:4353–61.
anti-TgCentrin1 (a gift from Pr. Gubbels, Boston College): Chen C-T, Kelly M, de Leon J, Nwagbara B, Ebbert P, Ferguson DJP, et al. Compartmentalized Toxoplasma EB1 bundles spindle microtubules to secure accurate chromosome segregation. Mol Biol Cell. 2015;26:4562–76.
anti-TgChromo1: Gissot M, Walker R, Delhaye S, Huot L, Hot D, Tomavo S. Toxoplasma gondii chromodomain protein 1 binds to heterochromatin and colocalises with centromeres and telomeres at the nuclear periphery. PloS One. 2012;7:e32671.
anti-TgSortilin: Sloves P-J, Delhaye S, Mouveaux T, Werkmeister E, Slomianny C, Hovasse A, et al. Toxoplasma sortilin-like receptor regulates protein transport and is essential for apical secretory organelle biogenesis and host infection. Cell Host Microbe. 2012;11:515–27.
anti-TgACP: Waller RF, Keeling PJ, Donald RGK, Striepen B, Handman E, Lang-Unnasch N, et al. Nuclear-encoded proteins target to the plastid in Toxoplasma gondii and Plasmodium falciparum. Proc Natl Acad Sci U S A. 1998;95:12352–7.
anti-TgTom40: van Dooren GG, Yeoh LM, Striepen B, McFadden GI. The Import of Proteins into the Mitochondrion of Toxoplasma gondii. J Biol Chem. 2016;291:19335–50.
anti-HA (Sigma-Aldrich; cat# 11867423001) : validated for Western-blots and IFA.
anti-myc (abcam; cat# ab10910) : validated for Western-blots and IFA.

ChIP-seq

Data deposition

☒ Confirm that both raw and final processed data have been deposited in a public database such as GEO.

☒ Confirm that you have deposited or provided access to graph files (e.g. BED files) for the called peaks.

Data access links
May remain private before publication.

ChIP-seq data have been desposited on GEO with the accession number GSE150406.
Go to https://www.ncbi.nlm.nih.gov/geo/query/acc.cgi?acc=GSE150406

Files in database submission

Input_AP2IX-5-1_S25_L002_R1_001.fastq
Input_AP2IX-5-2_S27_L002_R1_001.fastq
IP_AP2IX-5-1_S26_L002_R1_001.fastq
IP_AP2IX-5-2_S28_L002_R1_001.fastq
Input_AP2IX-5-1_S45_L008_R1_001.fastq
Input_AP2IX-5-2_S47_L008_R1_001.fastq
IP_AP2IX-5-1_S46_L008_R1_001.fastq
IP_AP2IX-5-2_S48_L008_R1_001.fastq
AP2IX-5.merged.cleaned.bowtie2_ME49.mark_duplicates.macs2_callpeak_extsize220_controlAP2IX5_peaks.narrowPeak

Genome browser session
(e.g. UCSC)

N/A

Methodology

Replicates

Two biological replicates were performed.

Sequencing depth

50bp single-reads were sequenced for Input and IP experiments (2 biological replicates each):
- Input/bio rep1: 7.5M raw reads, 6.3M cleaned reads, 4.8M aligned (4.4M uniquely aligned)
- Input/bio rep2: 7.3M raw reads, 6.1M cleaned reads, 4.5M aligned (4.1M uniquely aligned)
- IP/bio rep1: 8.1M raw reads, 6.8M cleaned reads, 2.6M aligned (2.4M uniquely aligned)
- IP/bio rep2: 4.9M raw reads, 4.1M cleaned reads, 1.8M aligned (1.6M uniquely aligned)

Antibodies

Rabbit IGg were use to perfom immunoprecipitation (Catalog Number A2909, Sigma-Aldrich)

Peak calling parameters

Mapping (for all experiments):
bowtie2 -x references/tgondii_ME49 -U input_reads.fq -S alignments.sam
Peak calling:
macs2 callpeak --bdg --nomodel --extsize 220 --gsize 65669794 --control input.bam --treatment ip.bam

Data quality

ChIP quality and datasets consistency was checked using the deepTools suite v3.1.3.

Software

Cleaned datasets (Input & IP * 2 biological replicates * 2 technical replicates = 8 datasets) were aligned with Bowtie2 v2.3.4 against

the T. gondii ME49 genome from ToxoDB-39 (Gajria et al., 2008) (14M aligned reads). Alignments from biological and technical replicates were merged using SAMtools v1.9 and duplicates were identified with Picard MarkDuplicates v2.18.20. ChIP quality and dataset consistency were checked using the deepTools suite v3.1.3 (Ramírez et al., 2016). Fragment size was estimated and peaks were called with MACS2 v2.1.2 (Zhang et al., 2008).
